# Deterministic Secure Quantum Communication on the BB84 System

**DOI:** 10.3390/e22111268

**Published:** 2020-11-07

**Authors:** Youn-Chang Jeong, Se-Wan Ji, Changho Hong, Hee Su Park, Jingak Jang

**Affiliations:** 1 The Affiliated Institute of Electronics and Telecommunications Research Institute, P.O.Box 1, Yuseong Daejeon 34188, Korea; w3140@nsr.re.kr (Y.-C.J.); sewanji@nsr.re.kr (S.-W.J.); hchc11@nsr.re.kr (C.H.); 2 Korea Research Institute of Standards and Science, Daejeon 43113, Korea; hspark@kriss.re.kr

**Keywords:** optical communication, quantum cryptography, quantum mechanics

## Abstract

In this paper, we propose a deterministic secure quantum communication (DSQC) protocol based on the BB84 system. We developed this protocol to include quantum entity authentication in the DSQC procedure. By first performing quantum entity authentication, it was possible to prevent third-party intervention. We demonstrate the security of the proposed protocol against the intercept-and-re-send attack and the entanglement-and-measure attack. Implementation of this protocol was demonstrated for quantum channels of various lengths. Especially, we propose the use of the multiple generation and shuffling method to prevent a loss of message in the experiment.

## 1. Introduction

By using the fundamental postulates of quantum mechanics, quantum cryptography ensures secure communication among legitimate users. Many quantum cryptography schemes have been developed since the introduction of the BB84 protocol in 1984 [1] by C. H. Bennett and G. Brassard. In the BB84 protocol, the sender uses a rectilinear or diagonal basis to encode information in single photons. To prevent interference by a malicious third party, the quantum no-cloning theorem (which asserts that a quantum state cannot be copied) is actively exploited. In the protocol, the bases are publicly announced by both the sender and the receiver who throw out the bits that were generated through mutually different bases. The remaining bits that share the bases are preserved. Then, Bob chooses a subset of these bits and sends it to Alice, who calculates the error rate to check whether the error rate value is within a certain threshold value. If this value exceeds the threshold, the intruder’s presence is revealed. Moreover, the Ekert protocol [2] is based on the non-locality of a Bell pair shared between users. If the measurement is performed by Alice and Bob using a compatible basis, sifted bits can be generated.

These quantum key distribution (QKD) schemes are not used to send message bits directly. They instead help establish a private key between Alice and Bob. After establishing the key, message bits are sent through a classical channel using the key and an encryption algorithm. To provide a simpler method, quantum secure direct communication (QSDC) schemes have been proposed. QSDC is used to communicate directly over a quantum channel without key generation steps [3]. QSDC systems thus enable one to send determined information aided by provable security based on the randomness of quantum mechanics. This means that secret messages can be delivered from sender to receiver without the classical communication from a ciphertext [4]. The first QSDC protocol was proposed by Long et al. [5] using the properties of entanglement and block transmission. In 2002, a ping-pong protocol published by Bostrom and Felbinger [6] drew attention because it allowed communication without any key by enabling Alice and Bob to directly send messages, as well as keys. Over the years, QSDC schemes using single photons and entangled states have been proposed [3,4,5,6,7,8,9,10,11,12,13,14,15,16,17,18,19,20,21,22]. Recently, Zhou et al. proposed device-independent QSDC (DI-QSDC) [23] based on device-independent QKD (DI-QKD) [24,25,26]. This DI-QSDC protocol uses the noiseless linear-amplification protocol [27] and the entanglement-purification protocol [28] to improve communication quality. Generally, QSDC needs two-quantum-state transmission to remove the need for classical information exchange, so this method can be complicated in terms of information exchange. In reality, one-quantum-state transmission is much more difficult and expensive than one classical information transmission.

Against this background, deterministic secure quantum communication (DSQC) is proposed. This method is analogous to, but a different form of, quantum communication. In this method, the transmitter encodes a secret message with the help of classical communication [29,30]. The difference between DSQC and QSDC is that DSQC needs classical information to decode the secret information in each photon, but QSDC does not. The first DSQC protocol was proposed by Beige et al. [29]. In this scheme, the receiver reads out the message only after the transmission of additional classical information (i.e., the cryptographic key of the sender). Subsequently, Li et al. suggested two DSQC protocols [31] based on pure entangled states and d-dimensional single-photon states. Lee et al. proposed a GHZ-state-based DSQC protocol [32] in which legitimate users could identify each other using the correlation of entangled states. Recently, Chang et al. and Yuan et al. proposed a DSQC protocol based on the W state and three qubit GHZ states, respectively [33,34]. Moreover, Li et al. proposed a DSQC protocol based on a GHZ-W state and a quantum one-time pad [35]. Jiang et al. suggested using DSQC in a single d-level system [30]. There are pre-sently many search results for studies on DSQC [29,30,31,32,33,34,35,36,37,38,39,40,41,42,43,44,45,46].

Here, we propose a DSQC protocol implementable on the BB84 frame work. The proposed protocol has several advantages. First, our protocol uses only a stream of single photons to ensure communication efficiency. The entangled-photon sources used in most DSQCs generally have a lower level of efficiency in practical implementations compared to single-photon sources. Secondly, our protocol applies entity authentication to increase security. In conversations that require security, verifying the identification of each user is a good way to prevent Eve from pretending to be a legal user. Unfortunately, unlike QKD, there is no known authentication method able to ensure the security of DSQC [47]. Therefore, we applied quantum entity authentication to identify legitimate users in real time rather than message authentication, which does not guarantee the uniqueness or timeliness of the data. Finally, we demonstrate the experimental feasibility of our protocol in a noisy channel. The security of our DSQC relies on the capability of users to detect eavesdropping during the security checking process. Moreover, the securely encoded messages in this scheme are not revealed to an attacker before the attacker is detected.

In an experiment, the channel loss of the photon is an inevitable reality. One must prepare for the possibility that the eavesdropper will conceal his or her presence and obtain information in the midst of losses. Thus, information leakage must be eliminated using privacy amplification, as in QKD. Unfortunately, privacy amplification is not available in DSQC and QSDC because it merges and mixes the original message. We suggest a viable alternative using the repetition and shuffling method, the security checking process, and quantum entity authentication.

The rest of the paper is organized as follows. In Section 2, our DSQC protocol is introduced. In Section 3, we discuss the security of the protocol against major attacks. In Section 4, we explain how we achieved our DSQC protocol with a heralded single-photon source and a polarization qubit. We summarize the results and offer conclusions in Section 5.

## 2. The Deterministic Secure Quantum Communication

In this section, we describe the details of our DSQC scheme. As with the BB84 protocol, Alice and Bob here use two bases for preparing and measuring: the $B_{z}$-basis ({|0〉, |1〉}) and the $B_{x}$-basis ({|+〉, |−}). The |0〉 and |+〉 states repre-sent message bit 0, and the |1〉 and |−〉 states repre-sent message bit 1. The detailed DSQC protocol is as follows.

The legitimate users, Alice and Bob, are connected through the quantum channel. The sender, Alice, prepares the initial state pair $\left({|ini\rangle }^{1},\;{|ini\rangle }^{2}\right)$. An initial state can be one of the four single states, |0〉,|1〉,|+〉, or |−〉. These four single states are used for many quantum communications, including the BB84 protocol. They are divided into two base groups, the $B_{z}$-basis ({|0〉, |1〉}) and $B_{x}$-basis ({|+〉, |−〉}). The mathematical relationships between the four single states are as follows:

(1) $$|0\rangle =\frac{1}{\sqrt{2}}\left(|+\rangle +|-\rangle \right),\;|1\rangle =\frac{1}{\sqrt{2}}\left(|+\rangle -|-\rangle \right)\;|+\rangle =\frac{1}{\sqrt{2}}\left(|0\rangle +|1\rangle \right),\;|-\rangle =\frac{1}{\sqrt{2}}\left(|0\rangle -|1\rangle \right).$$

The initial state pairs are composed of the message source pairs and the checking source pairs. For message transmission, Alice chooses the message source pairs. She uses the checking source pairs to verify the security of the quantum channel and authenticate the legitimate users.

For quantum entity authentication, the legitimate users pre-share the authentication key, $K_{AB}=\left(k_{AB1},k_{AB2},\ldots ,k_{ABu}\right)$. The length of the authentication key is u bits. The procedures are as below.

### Protocol

Step 0. The message sender, Alice, prepares message M to send. The message M consists of a sequence of m bits. Alice randomly shuffles the locations of each bit that make up the message M to form a new sequence $M^{’}$ and stores information about the mixes.
Step 1. Alice randomly chooses the message process and the security checking process. She chooses the security checking process with probability C. The sender selects the message process with the remaining probability, 1−C. If Alice chooses the message process, proceed to Step M1. If not, move to Step S1.
Step M1. Alice prepares a message source pair ${\left({|ini\rangle }^{1},\;{|ini\rangle }^{2}\right)}_{i}$ according to a bit of the shuffled message $M^{’}$. Alice randomly prepares a state pair $\left({|0\rangle }^{1},\;{|0\rangle }^{2}\right)$ or $\left({|+\rangle }^{1},\;{|+\rangle }^{2}\right)$ for which the bit value is 0. Similarly, she randomly prepares $\left({|1\rangle }^{1},\;{|1\rangle }^{2}\right)$ or $\left({|-\rangle }^{1},\;{|-\rangle }^{2}\right)$ for message bit 1. For example, she can create the message source pair ${\left({|ini\rangle }^{1},\;{|ini\rangle }^{2}\right)}_{i}={\left({|+\rangle }^{1},\;{|+\rangle }^{2}\right)}_{i}$ for message 0. Here, i is the number of the locations of all n pairs. We assume that the total DSQC process will be iterated n times.
Step M2. Alice sends Bob the message source pair $\left({|ini\rangle }^{1},\;{|ini\rangle }^{2}\right)$ through the quantum channel.
Step M3. Bob randomly chooses one measurement basis pairs of $\left(B_{z},B_{x}\right)$ and $\left(B_{x},B_{z}\right)$. $\left(B_{z},B_{x}\right)$ means that ${|ini\rangle }^{1}$ and ${|ini\rangle }^{2}$ are measured on the $B_{z}$ and $B_{x}$ bases, respectively. Similarly, the expression $\left(B_{x},B_{z}\right)$ indicates that ${|ini\rangle }^{1}$ and ${|ini\rangle }^{2}$ are measured with the $B_{x}$ and $B_{z}$ bases, respectively.
Step M4. Bob measures the received message source pair with the measurement basis sequence selected in the previous step M3. For convenience, let us annotate the measurement result pair of location i as ${\left(O_{1},O_{2}\right)}_{i}$, where $O_{1}\left(O_{2}\right)\in \left\{0,1,+,-\right\}$. Thus, there is a total of eight possible result pairs. If i<n, go to Step 1. Here, n is total number of pairs used in our protocol. However, if the length of the intended message, m, is not met (even if i=n is satisfied), then go to Step 1. Here, m and n have the relationship of m<n. If i=n, and the message length m is satisfied, move to Step 2.
Step S1. Alice generates a checking source pair ${\left({|chk\rangle }^{1},\;{|chk\rangle }^{2}\right)}_{j}$, where ${|chk\rangle }^{1,2}\in \left\{|0\rangle ,|1\rangle ,|+\rangle ,\;|-\rangle \right\}$. The total number of possible checking source pairs is 16. Unlike the message source pair, these pairs can be organized with different bases to form a pair so that (|0〉,|+),(|0〉,|−〉),(|1〉,|+),(|1〉,|−),(|+〉,|0〉), (|+〉,|1〉), (|−〉,|0), and (|−〉,|1〉) are possible for the checking source pairs. It is possible to configure a pair in different states or in the same quantum state. These pairs are (|0〉,|0〉),(|0〉,|1〉),(|1〉,|0〉),(|1〉,|1〉), (|+〉,|+〉),(|+〉,|−〉),(|−〉,|+〉), and (|−〉,|−〉).
Step S2. Alice transmits Bob the checking source pair ${\left({|chk\rangle }^{1},\;{|chk\rangle }^{2}\right)}_{j}$ using the quantum channel.
Step S3. Similar to Step M3, Bob makes a measurement on the received pair with the chosen measurement basis pair ($\left\{B_{z},B_{x}\right\}$ or $\left\{B_{x},B_{z}\right\}$). As previously stated, the measurement outcomes are expressed in ${\left(O_{1},O_{2}\right)}_{j}$. Bob then publicly announces that he has received a pair and measured it.
Step S4. Alice selects either the authentication mode or the security confirmation mode with the specified probability. In other words, Alice chooses the authentication mode with the probability $a_{u}C$ and the security confirmation mode with the probability $\left(1-a_{u}\right)C$. At this time, the process must be iterated from step 1 to the current step until the number of pairs of the authentication mode is u. Alice securely records the mode of her own choosing. Go to Step 1.
Step 2. Bob publicly announces to Alice that he has received all pairs.
Step 3. Alice notifies Bob of the positions of the authentication mode.
Step 4. After Alice’s announcement of the location of the authentication mode, Bob calculates
  (2) $${\left(Au_{1},\;Au_{2}\right)}_{r}\;=\;{\left(O_{1}^{j}⊕k_{AB\;r},\;O_{2}^{j}⊕k_{AB\;r}\right)}_{r}$$
  where j is the location of the authentication mode, and $k_{AB\;r}$ is the entity of the pre-shared authentication key $K_{AB}$. For convenience, the operator ⊕ is defined as the qubit flip operator as follows:
  (3) 0 ⊕ 0≡0, 0 ⊕ 1≡1, 1 ⊕ 0≡1, 1 ⊕ 1≡0+ ⊕ + ≡+, + ⊕ − ≡−,− ⊕ + ≡−,− ⊕ − ≡+.
Step 5. Bob tells Alice ${\left(Au_{1},\;Au_{2}\right)}_{r}$, where r=1, 2,…, u. Then, Alice can verify Bob’s identification as follows. Alice knows the initial states ${\left({|ini\rangle }^{1},\;{|ini\rangle }^{2}\right)}_{r}$, the authentication key $K_{AB}$, Bob’s measurement basis information ($\left(B_{z},B_{x}\right)$ or $\left(B_{z},B_{x}\right)$), and the measurement results ${\left(O_{1},O_{2}\right)}_{r}$. Alice takes the information that she and Bob have computed on the same basis from ${\left(Au_{1},\;Au_{2}\right)}_{r}$. Alice checks whether the chosen $Au_{1\;or\;2}$ is ${\left(chk\right)}_{r}^{1\;or\;2}⊕k_{AB\;r}$. For example, Alice prepares and sends ${\left({|chk\rangle }^{1},\;{|chk\rangle }^{2}\right)}_{r}={\left({|1\rangle }^{1},{|0\rangle }^{2}\right)}_{j}$, and then Bob measures the received state pair with $\left(B_{z},B_{x}\right)$. If his measurement outcomes are ${\left(1^{1},\;{+}^{2}\right)}_{r},$ and $k_{AB\;r}=1$, then Bob announces that ${\left(Au_{1},\;Au_{2}\right)}_{r}=\left(0^{1},{-}^{2}\right)$. After listening to Bob, Alice selects $Au_{1}=0^{1}$ because it is the same as the basis of the prepared state ${|chk\rangle }_{r}^{1}$ ($B_{z}$-basis). Alice then checks whether $Au_{1}={\left(chk\right)}_{r}^{1}⊕k_{AB\;r}$. Alice also determines whether Bob possesses an authentication key $k_{AB\;r}$. Alice and Bob perform the above-described procedure on a total of u pairs prepared as the authentication states, ${\left(Au_{1},\;Au_{2}\right)}_{r}$. Note that the probability of pairs not being authenticated among the u pairs is 1/4. Because half of the states prepared by Alice as the authentication state are prepared with different bases (e.g.,$\;\left(B_{z},B_{x}\right)$), the probability that, for the different bases, the measurement basis pair $\left(B_{z},B_{x}\right)$ or $\left(B_{x},B_{z}\right)$ selected by Bob will be identical to the prepared bases is 1/2. If the bases are not identical, authentication cannot be performed. Given the probability of the pairs not being authenticated, if the authentication procedure is successful, proceed to the next step.
Step 6. Alice announces to Bob the positions of the security confirmation mode.
Step 7. Bop reveals the outcomes of the measurement at the location of the qubit pair corresponding to the security confirmation mode.
Step 8. Alice checks the security of the quantum channel as follows. She compares the results of the measurement performed using the same basis as her initial basis and the initial state, ${\left({|chk\rangle }^{1},\;{|chk\rangle }^{2}\right)}_{k}$, where k=1,2,…, l. Similar to the authentication mode, there is a probability of 1/4 that the measurement will not be checked. If the security confirmation mode is successful, go to the next step.
Step 9. After the authentication mode and the security confirmation mode have been carried out successfully, the following procedure is performed. For the remaining pair, Alice announces her chosen basis pair (the message-source pair) and the shuffling information to Bob.
Step 10. Bob can decode the message using Alice’s announced basis information and reorder his information and outcomes measured on the same basis as those of Alice.

**Figure 1:**
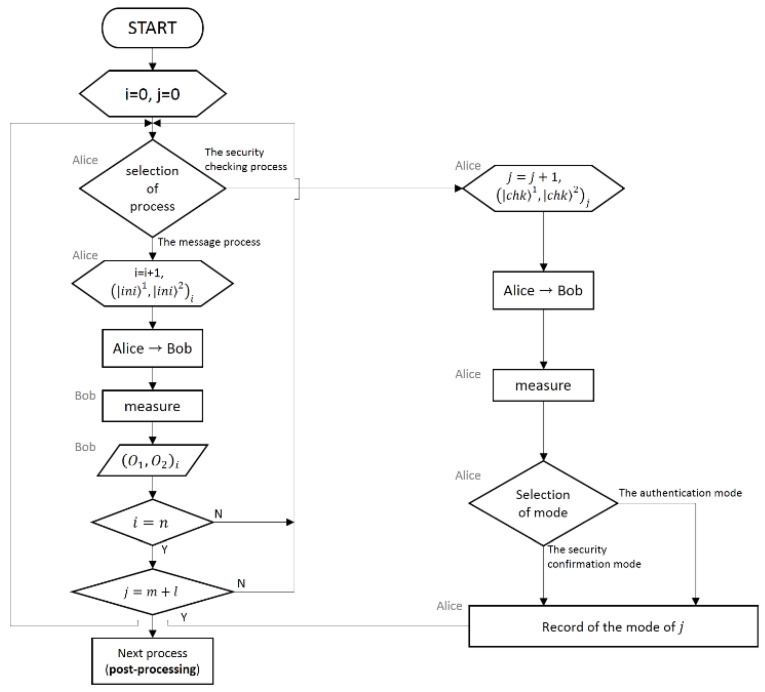
Flowchart of our deterministic secure quantum communication protocol (except for post-processing).

**Figure 2:**
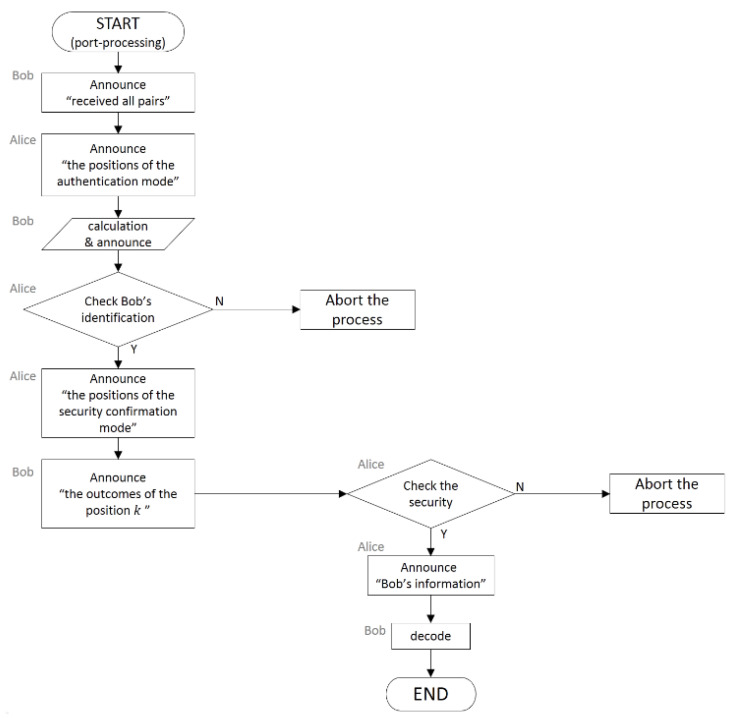
Flowchart of the post-processing of the deterministic secure quantum communication protocol.

The process of the protocol can be pictorialized as the flowcharts pre-sented in Figure 1 and Figure 2.

## 3. Security Discussion

We next discuss the security of our DSQC protocol against an intercept-and-re-send attack and an entanglement-and-measure attack. In the intercept-and-re-send attack, an eavesdropper (Eve) captures the sending qubits from Alice and sends her own qubits to Bob. In this case, the probability of the attacker’s detection from the respective qubit pairs used in the authentication mode and in the security confirmation mode are described below. The total number of qubit-pair types used in the authentication states and the security confirmation states is 16.

Suppose Alice transmits quantum states (|0〉,|0〉) of the 16 pairs. This may be equally applied to the case where Alice transmits (|1〉, |1〉), (|+〉, |+〉), and (|−〉, |−〉). When these states are intercepted by Eve in the middle of the communication, the measurement basis pair, $\left(B_{x},B_{z}\right)$, is selected between basis pairs $\left(B_{z},\;B_{x}\right)$ and $\left(B_{x},B_{z}\right)$. Because the second quantum state |0〉 measured using the $B_{z}$- basis by Eve is prepared with the same basis used by Alice, the result of the measurement by Eve becomes |0〉. In contrast, because Eve measures the first quantum state on the $B_{x}$- basis, which is different from that of Alice, the quantum state might be re-sent to Bob at a probability of 1/2. The situation in which the quantum state will not be re-sent at a probability of 1/2 is if the results of decoding the first measurement result and the second measurement result are not identical to each other. This case corresponds the measurement results of (|1〉,|0〉), for example. The case where Eve re-sends the quantum states is a situation where the results of the measurement are (|+〉, |0〉). Bob may receive the quantum state pair (|+〉, |0〉) and randomly select one pair between $\left(B_{z},\;B_{x}\right)$ and $\left(B_{x},B_{z}\right)$ with a probability of 1/2 and then measure the quantum state pair. In this case, when Bob selects the basis pair $\left(B_{x},B_{z}\right)$, the presence of Eve cannot be detected; however, when Bob selects the basis pair $\left(B_{z},\;B_{x}\right)$, the presence of Eve can be detected at a probability of 1/2. In the entire process, the probability that Bob will detect Eve is 1/8. When the remaining 12 pairs are also analyzed using a method similar to that described above, a detection probability of 1/8 is obtained for each of the pairs. That is, when a total of u+l authentication and security confirmation pairs are used, the probability that Eve will be detected is

(4) $$1-{\left(1-\frac{1}{8}\right)}^{u+l}.$$

When u+l is sufficiently large, the probability approaches 1. That is, through a sufficient number of authentication and verification pairs, Eve can be detected with 100% certainty. For security, when Eve is detected in advance by a sufficiently increasing u, which is the number of authentication pairs, the leakage of messages can be minimized.

Next, we analyze the security of our DSQC protocol against an entangle and measure attack. In this attack, to gain Alice’s message information, the eavesdropper first performs the attack operation $\hat{A}$ on the transmitting quantum state (the state of the message source pair or checking source pair) with her auxiliary state |ω〉. Then, Eve lets the sending state go to the receiver, Bob. After the transmission procedure, Eve measures her auxiliary state and tries to obtain information about the message using the measurement outcomes. To analyze Eve’s strategy, we must define the attack operation $\hat{A}$, which can be written as

(5) $$\hat{A}|0,w\rangle =\alpha |0,w_{00}\rangle +\beta |1,w_{01}\rangle$$

(6) $$\hat{A}|1,w\rangle =\beta |0,w_{10}\rangle +\alpha |1,w_{11}\rangle$$

and

(7) $$\hat{A}|+,w\rangle =\frac{1}{2}|+\rangle \left[\alpha |w_{00}\rangle +\beta |w_{01}\rangle +\beta |w_{10}\rangle +\alpha |w_{11}\rangle \right]\;+\;\frac{1}{2}|-\rangle \left[\alpha |w_{00}\rangle -\beta |w_{01}\rangle +\beta |w_{10}\rangle -\alpha |w_{11}\rangle \right]$$

(8) $$\hat{A}|-,w\rangle =\frac{1}{2}|+\rangle \left[\alpha |w_{00}\rangle +\beta |w_{01}\rangle -\beta |w_{10}\rangle -\alpha |w_{11}\rangle \right]\;+\;\frac{1}{2}|-\rangle \left[\alpha |w_{00}\rangle -\beta |w_{01}\rangle -\beta |w_{10}\rangle +\alpha |w_{11}\rangle \right]$$

where ${\left|\alpha \right|}^{2}+{\left|\beta \right|}^{2}=1$ and $\langle w_{00}|w_{10}\rangle +\langle w_{01}|w_{11}\rangle =0$.

To ease the security discussion, let $\langle w_{00}|w_{01}\rangle =\langle w_{10}|w_{11}\rangle =\langle w_{00}|w_{10}\rangle =\langle w_{01}|w_{11}\rangle =0$. Assume that Alice sends the |0〉 state prepared as the state of the checking source pair. Then, the state received by Bob is

(9) $$|\psi^{0}\rangle =\hat{A}|0,w\rangle =\alpha |0,w_{00}\rangle +\beta |1,w_{01}\rangle .$$

Using Equation (9), the eavesdropper is detected with a probability of

(10) $$p^{d}\left(|0\rangle \right)={\left|\beta \right|}^{2}.$$

If Alice’s transmitting state is |1〉, the calculated detection probability is

(11) $$p^{d}\left(|1\rangle \right)={\left|\beta \right|}^{2},$$

as above. Next, suppose that the traveling state is |+〉, prepared as the state of the checking source pair and that, in this case, Bob’s received state is

(12) $$|\psi^{+}\rangle =\hat{A}|+,w\rangle \;=\frac{1}{2}|+\rangle \left[\alpha |w_{00}\rangle +\beta |w_{01}\rangle +\beta |w_{10}\rangle +\alpha |w_{11}\rangle \right]\;+\;\frac{1}{2}|-\rangle \left[\alpha |w_{00}\rangle -\beta |w_{01}\rangle +\beta |w_{10}\rangle -\alpha |w_{11}\rangle \right].$$

Then, the probability of detecting the eavesdropper is

(13) $$p^{d}\left(|+\rangle \right)=1-\left\{\frac{1}{2}\times \frac{1}{4}\left({\left|\alpha \right|}^{2}+{\left|\beta \right|}^{2}+{\left|\beta \right|}^{2}+{\left|\alpha \right|}^{2}\right)\right\}=\frac{1}{4}.$$

In subsequent calculations, the detection probability of Equation (13) is also satisfied when the transmitting state is |−〉. Therefore,

(14) $$p^{d}\left(|-\rangle \right)=1-\left\{\frac{1}{2}\times \frac{1}{4}\left({\left|\alpha \right|}^{2}+{\left|\beta \right|}^{2}+{\left|\beta \right|}^{2}+{\left|\alpha \right|}^{2}\right)\right\}=\frac{1}{4}.$$

In combination, the total probability of detecting Eve is expressed as

(15) $$p^{d}=\frac{1}{2}\left[{\left|\beta \right|}^{2}+\frac{1}{4}\right].$$

Notably the total detection probability, $p^{d}$, is a function of β only. This means that Eve will try to make β as small as possible to minimize her detection probability. However, she cannot configure the operator $\hat{A}$ that makes β=0 because the sending source pair selection (in Step M1 or Step S1) is unknown. Even if Eve sets β=0, detection is still possible with a probability $p^{d}=1/8$.

Next, let us analyze the amount of information Eve could maximally gain if no security checking procedure were executed. Consider the case where Alice sends |0〉. After her attack operation, the state of the system reads

(16) $$\rho^{\prime }={\left|\alpha \right|}^{2}\;|0,w_{00}\rangle \langle 0,w_{00}|+{\left|\beta \right|}^{2}|1,w_{01}\rangle \langle 1,w_{01}|\;+\alpha \beta *\;|0,w_{00}\rangle \langle 1,w_{01}|+\alpha *\beta \;|1,w_{01}\rangle \langle 0,w_{00}|$$

(17) $$=\left(\begin{array}{cc} {\left|\alpha \right|}^{2} & \alpha \beta *\\ \alpha *\beta & {\left|\beta \right|}^{2} \end{array}\right),$$

where, on an orthogonal basis $\left\{|0,w_{00}\rangle ,\;|1,w_{01}\rangle \right\}$, the maximal information $I_{0}$ can be obtained from Equation (17) as

(18) $$I_{0}=S\left(\rho^{\prime }\right)=-Tr\left(\rho^{\prime }\log_{2}\rho^{\prime }\right)\;=-\lambda_{1}log_{2}\lambda_{1}-\lambda_{2}log_{2}\lambda_{2}.$$

Therefore, to calculate the von Neumann entropy, eigenvalues $\lambda_{1}$ and $\lambda_{2}$ are needed. These eigenvalues are

(19) $$\lambda_{1,2}=\frac{1}{2}\left[1\pm \sqrt{1-8{\left|\beta \right|}^{2}\left\{{\left|\beta \right|}^{2}-1\right\}}\right].$$

When ${\left|\beta \right|}^{2}=0$, namely, if Eve chooses attack operator $\hat{A}$ that provides the minimum detection probability $p^{d}=1/8$, the maximal information $I_{0}=0$. This implies that Eve obtains no information at all. The detection probability $p^{d}$ increases dramatically according to the information gains of Eve. The same discussion applies equally to the other cases. If Eve chooses operator $\hat{A}$ to minimize her exposure to legitimate users, the overall detection probability $p^{d}$ is 5/8, as per Equation (15). By using a sufficient number of checking source pairs, intermediate attackers can be detected with 100% probability. Therefore, our DSQC protocol is secure against a entangled and measure attack.

## 4. Experimental Implementation

In practical situations, message information loss is unavoidable because we use photons to communicate messages through lossy optical fibers or free space. To prevent the loss of message information, we propose a multiple generation and modified shuffling method for implementing step 0. The sender (Alice) prepares multiple photons using quantum states similar to a repetition code. For example, to encode classical bit 0, Alice prepares the same 20 quantum states as ${|0\rangle }^{⊕n}⊕{|+\rangle }^{⊕m},(n+m=20)$. These multiple states basically allow the eavesdropper (Eve) to obtain a great deal of information about the message. To avoid this, Alice randomly shuffles or rearranges these multiple photons with other multiple photons comprising other message bits and records the way that the photons are shuffled. With our DSQC protocol, two consecutive qubits are sent to deliver one classical bit, and the shuffling does not change this. After Bob receives the stream of photons, he reveals the arrival time of the photons before receiving the measurement axis information from Alice. Then, Alice announces the correct order of the photons, which are used to decode the message. If Bob obtains more than two two-consecutive photon pairs that correspond to the same classical bit, Alice announces only one pair of them. To implement this multiple generation and shuffling protocol in a real experiment, Alice generates multiple classical messages and shuffles them. These reordered classical messages look like a random sequence. Alice should then record the order in which the messages are shuffled. Then, following this reordered sequence, Alice generates a pair of photon states randomly. Since privacy amplification, unlike QKD, is not possible in our DSQC, to secure communication, the communication of messages must proceed after the entity (user) authentication succeeds, and the presence of the attacker is confirmed.

**Figure 3:**
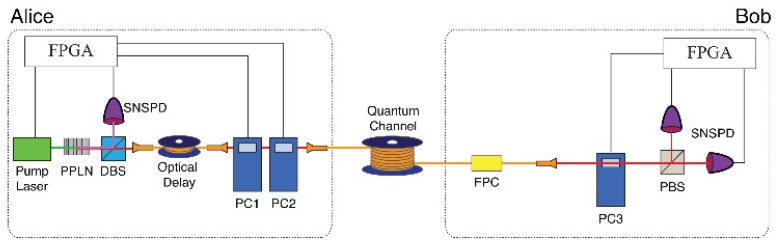
Experimental setup. Alice: The heralded single-photon source consists of a pump laser, PPLN, dichroic beam splitter (DBS), and superconductor nanowire single photon detector (SNSPD). The polarization qubits of single photons are encoded by two Pockels cells (PC1, PC2). Bob: The change of polarization in the fiber is compensated by the fiber polarization controller (FPC). The polarization state is decoded by a Pockels cell (PC3), polarizing beam splitter (PBS), and two SNSPDs.

We next demonstrate the proposed DSQC protocol using a heralded single-photon source and polarization qubits. The schematic of our DSQC system is shown in Figure 3. Alice encodes a polarization state into a single photon generated by spontaneous parametric down-conversion (SPDC) in a 10 mm-long type-I PPLN crystal, pumped by a 520-nm mode-locked laser (40 MHz repetition rate). The PPLN crystal emits 782 nm idler photons and 1552 nm signal photons, and the idler photons are detected by a trigger superconductor nanowire single photon detector (SNSPD). The SNSPD has >80% detector efficiency and less than a 100 Hz dark count at 1550 nm. The polarization state of the 1552 nm signal photon is encoded by two Pockels cells. Bob receives the signal photon after transmission through a single mode fiber (SMF28e) line. The measurement basis is randomly chosen by one Pockels cell using either the $B_{x}$ or $B_{z}$ basis along with a polarization analyzer and two SNSPDs. For Alice and Bob, the data process and Pockels-cell driving signals are separately controlled by a field-programmable gate array module (FPGA). The heralding efficiency of the heralded single-photon source is 45% for 1552 nm signal photons, for which the count rate is 400 kHz. The second-order correlation of the heralded single photons is $g^{2}\left(0\right)\approx 0.045$.

The light generated using SPDC also generates double pairs that could be exploited in PNS attacks. However, the heralded single photon source (HSP) based on SPDC has much lower probability for double pair generation compared with the attenuated laser ($g^{2}\left(0\right)=1$). The second order correlation $g^{2}\left(0\right)$ is about 0.048 for our HSP.

**Figure 4:**
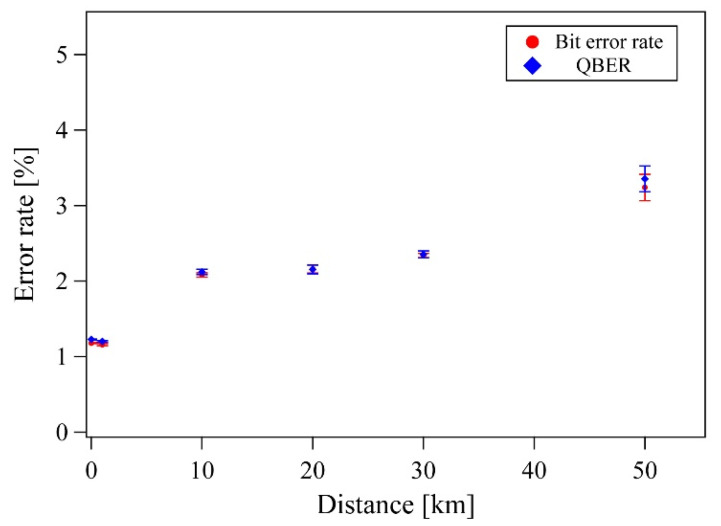
Bit error rate (BER) and quantum bit error rate (QBER). The BER is the error bit over the total message size in the message mode. The QBER is the error bit over the sifted key in the control mode. (“Bit error rate” → “BER” in the figure).

**Figure 5:**
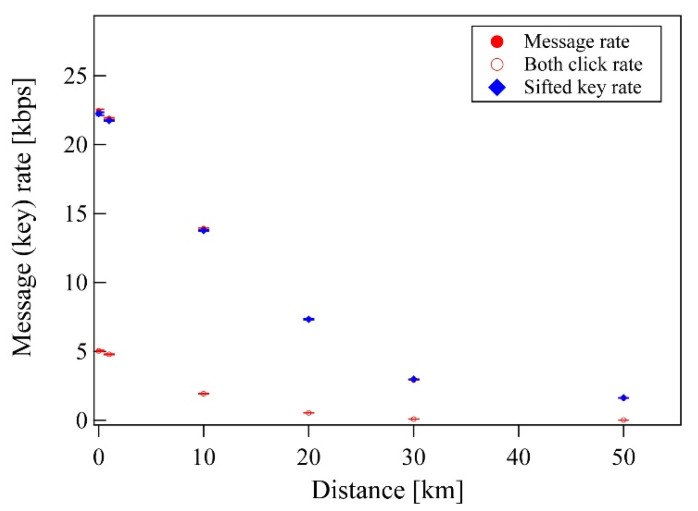
Message and key rate for various distances. The message rate is the average number of transmitted bits per one second in the message mode. In the message mode, dual clicks contribute to the count rate when Bob detects two qubits for one-bit message. Sifted keys are keys generated in the control mode.

We next experimentally investigated the proposed DSQC protocol for various quantum channel lengths. In this experiment, Alice sent a 655,360-qubit train to Bob, and then Alice randomly selected message modes with 50% probability. In the message mode, Alice encoded pre-processed data bits into polarization qubits after their conversion from message bits, considering the subsequent error correction. The remaining 50% probability was in the control mode and confirmed the security of the DSQC system via the BB84 protocol. The control mode used the same procedures as the BB84 quantum key distribution protocol. Its sifted key rate was 15 kbps with a 10 km single-mode fiber link, and the QBER was about 2% (Figure 4 and Figure 5). The QBER is related to a variety of factors, including detector noise, optical misalignments, polarization mode dispersion, Raman scattering, and so on. In this experiment, the QBER was affected by the optical axis mismatch between several wave plates (not shown in Figure 3) used for high-speed polarization encoding/decoding with Pockels cells (PC1, PC2, and PC3) in free space. In the message mode, the data rate was 15 kbps with the 10 km fiber. This is the same rate as the sifted key rate in the control mode and is reasonable because Alice and Bob have a 50% probability of sharing the basis, as Alice randomly selected $B_{x}$-$B_{z}$ or $B_{z}$-$B_{x}$ bases and Bob measured $B_{x}$-$B_{x}$ or - bases.

## 5. Conclusions

The proposed DSQC protocol based on the BB84 system has been proven theoretically to be resistant to major attacks from eavesdropping, for instance, via the intercept-re-send attack and the entangle-and-measure attack. Although eavesdropping disturbs communication between users, at the same time, the probability of detecting eavesdropping is also enhanced. Moreover, Alice and Bob identify themselves before sending secure messages to prevent Eve’s intervention. The shuffling method introduced in our protocol has the specific advantage of being able to cope with photon losses during its implementation. The main advantage of our DSQC protocol is that it can be implemented to include quantum entity authentication in the BB84 system. Recently, QKD was commercialized [48,49]. By developing a DSQC protocol based on the BB84 scheme, which is the pivotal element of QKD, here, we lay the foundation for progress on the DSQC protocol beyond the laboratory level. It is noteworthy that QKDs needs a policy to manage the generated key. There is a time delay between key generation and key use because the key is used when communication is needed rather than immediately upon generation. Therefore, generated keys have to be stored safely, and clear procedures for their use have to be specified. DSQCs do not require a key-management policy because secure communication is possible without a key. Sending secure messages without keys is, moreover, a completely new technology. At this point, however, there are some significant elements that must be studied and solved in the DSQC protocols. The unconditional security of DSQC has not yet been proven, even though some researchers are trying to prove information-theoretical security. Since privacy amplification is not yet available in most DSQCs [31,32,33,34,35,36,37,38,39,40,41,42,43,44,45,46], including our DSQC, further studies should focus how to supplement information leakages. Moreover, the DSQC protocols, for which classical communication is important, cannot be guaranteed to be more efficient than BB84 followed by one-time padding. Therefore, further studies on DSQC are needed. As with early research on QKD, the DSQC protocol also needs a process of maturation based on a variety of approaches. In that sense, creating our DSQC within the BB84 system can be understood as part of the efforts to enhance DSQC’s feasibility.
